# Supplementary material for: “Talking on the Phone Is Very Cold”—Primary Health Care Nurses’ Approach to Enabling Patient Participation in the Context of Chronic Diseases during the COVID-19 Pandemic
Source: Healthcare (Basel). 2022 Dec 2;10(12):2436. doi: 10.3390/healthcare10122436 (PMC9777830; doi:10.3390/healthcare10122436)
Supplement: Supplementary file 1 [file healthcare-10-02436-s001.zip › healthcare-2030481-supplementary.pdf]

Guideline for interviews with nurses Phase 1

In our study we analyze how family and community involve patients in their care. We conduct this study in Germany, Spain and Brazil.

More specifically, we are interested in how do nurses (a) work with patients and their families individually, and (b) how they work with groups and communities in order to promote health and to address the needs of people with chronic diseases.

We will interview several experts in primary health care and nursing, like directors or coordinators in primary health care and health centers. And of course, we interview nurses who work in direct patient care.

I will ask you several questions, for example on your tasks in certain areas and how you involve users in their care. If I ask questions that you are not able to or you do not want to answer, please let me know. I cannot always estimate how familiar the different interview partners are with the topics. We are asking the same questions in all of the three countries; however, we know that nurses in different countries have different profiles and because of that can tell us more or less according to the different questions.

Introduction

1. At the beginning could you please introduce yourself? What is your education? What is your occupational position and what are your current tasks.

Tasks of family and community nurses; task division and collaboration with doctors (other health professionals) in chronic care

2. Could you give me an overview on the main tasks of the nurse(s) in your health center(s)

   a) Could you tell me more in detail? Which tasks do you assume in the area of health promotion? Could you describe a – in your view – typical activity in health promotion, that illustrates how you act?

   b) Could you tell me more in detail: Which tasks do you assume in the care of chronically ill patients/users? Could you tell me a case example of a chronically ill patient that - in your view – shows which tasks you typically perform in the care of chronically ill patients?

      Compared to your colleagues, do you perform special or other tasks in the care for chronically ill patients? What are these tasks?

   c) If you look at the last years in your health center/family health team, have there been changes in your activities in the care for chronically ill patients? Could you please describe these changes for me.

3. How is the division of tasks between you and the doctor(s) in caring for chronically ill people designed? Could you please describe for me how the task division looks like?

a) *Afterthought:* Could you give me please a “typical” case example for this task division?

b) In your opinion, what is going well in the collaboration with the doctor(s) in the care for chronically ill patients? And vice versa: What could be better in this collaboration? (Can you tell me a situation so that I can imagine it better)

Individual participation of people with chronic diseases and their families in the care as a responsibility for nurses

Now I would like to ask you questions about how you involve people with chronic diseases and their families in their care.

4. How do you strengthen the competencies of your patients so that they can better deal with their own illness? Can you please tell me about such approaches?

5. When you think of the everyday life of your chronically ill patients/users: How do you make it easier for your patients/clients to live with the illness in everyday life? Can you give me a case example?

6. How/in which way do you involve patients/users in decisions regarding their own care? Could you tell me that by using an example or a situation?

7. Are there also situations where the "freedom" of your patients to co-decide on their care reaches its limits? Can you tell me about such a situation?

8. How do you cooperate with family members of your chronically ill patients? Can you tell me a case example? Is there anything that makes it easier or more difficult for you to involve family members?

9. If you think of different population groups, e.g., different age groups, social classes, people with certain chronic diseases: Do you see any differences in whether and how these different groups can be or want to be involved in their care? Could you give an example?

10. In your experience, what working conditions in your health center make it easier for you to promote the involvement of your patients/users in their care?
(Afterthought: What are other working conditions that facilitate you user participation in their care) (Examples: time/workload)

    a. And vice versa, which working conditions make it more difficult for you to promote the participation of the users in their care?
    (Afterthought: Can you name further working conditions that have proven to be unfavorable...?)

11. Do or did you attend training or education, or do you receive supervision that helps you to involve patients and relatives in their care? If so, could you tell me more on what they contain?

Promotion of participation in groups and participation of communities as a responsibility of nurses

We are also interested in whether and how you work with patient/user groups and with communities.

12. Do you work with patient/user groups? Which groups are that and what are your tasks when you work with these groups?

    a. *If question 12 "yes":* In the work with patient groups, how do you strengthen the participation of the people in the group?
    In case of a possible 'misunderstanding' of IP: Let me better explicate the form of participation that I mean: How promote you the active engagement of the people during the group meetings, for example that the group members share their experiences in the group

    b. Could you tell me about difficulties that you face when you would like to promote participation of patients/users during the group meetings?

    c. What supports you in your ability to work with groups? Can you tell me an example or a situation?

13. Do you work in other community settings, e.g. in schools, youth centres, senior centres, and others? If so, in which way do you promote the participation of citizens in community health issues? (Could you give an example?)

14. Are you involved in further projects or initiatives that have the objective to identify needs or problems of the community? Could you tell me more about what you are doing?

15. What factors are supporting (could support) your commitment in community participation, which factors are hindering it?

Overall assessment on the relevance; facilitating and hindering conditions for strengthening user participation by nurses

16. Overall, how relevant is user participation in your daily work?

17. Overall, which are the most important conditions that strengthen you in your daily work to practically implement a greater participation of the users?

18. And vice versa: Which conditions hinder you to practically implement a greater participation of the users?

    Thank you very much for your attention and for the interesting interview, I have asked all my questions. Is there something that you would like to add?

Guideline for interviews with nurses Phase 2

In our study we analyze how family and community nurses involve patients/users in their care during the COVID-19 Pandemic.

More specifically, we are interested in nurses (a) work with patients and their families individually, and (b) with groups and communities.

We are interested in how your work with patients, families, groups, and communities changed during the pandemic and the in factors that facilitated or complicated your work.

I will ask you several questions, for example on your tasks in certain areas and how you involve users in their care. If I ask questions that you are not able to or you do not want to answer, please let me know. I cannot always estimate how familiar the different interview partners are with the topics.

In this interview I am only interested in your personal opinions; there are no wrong or right answers.

Introduction

1. At the beginning could you please introduce yourself? What is your education? What is your occupational position and what are your current tasks?

Tasks of family and community nurses during the COVID-19 pandemic

2. Could you give me an overview on the main tasks of the nurse(s) in your health center(s) during the COVID-19 pandemic and furthermore? How did these tasks change due to the COVID-19 Pandemic

   a) Could you tell me more in detail: Which tasks do you assume in the care for chronically ill patients/users at the moment? Could you tell me a case example of a chronically ill patient that - in your view – shows which tasks you typically perform in the care of chronically ill patients?

   b) If you look at the last two years in your health center, how did your activities in the care for chronically ill patients change during the pandemic? Could you please describe these changes for me?

Individual participation of people with chronic diseases and their families during the COVID-19 pandemic as a responsibility for nurses

Now I would like to ask you questions about how you involve people with chronic diseases and their families in their care during the COVID-19 pandemic.

3. How do you strengthen the competencies of your patients so that they can deal with their own illness better during the pandemic? Can you please tell me about your approaches?

4. How do you involve patients/users in decisions regarding their own care? How did the way you involve patients in their own care change during the pandemic? Could you tell me that by using

an example or a situation?

5. How do you cooperate with family members of your chronically ill patients during the COVID-19 pandemic? Can you tell me a case example? Is there anything that makes it easier or more difficult for you to involve family members during the pandemic?

6. If you think of different population groups, e.g. different age groups, social classes, people with certain chronic diseases: Do you see any differences in whether and how these different groups can be or want to be involved in their care during the COVID-19 pandemic? Could you give an example?

7. In your experience, what working conditions in your health center make it easier for you to promote the involvement of your patients/users in their care during the COVID-19 pandemic? (Afterthought: What are other working conditions that facilitate your activities in enabling users‘ participation in their care) (Examples: time/workload)

    a. And vice versa, which working conditions make it more difficult for you to promote the participation of the users in their care during the COVID-19 pandemic? (Afterthought: Can you name further working conditions that have proven to be unfavorable...?)

Promotion of participation in groups and participation of communities as a responsibility of nurses

We are also interested in whether and how you work with patient/user groups and with communities during the COVID-19 pandemic.

8. Do you work with patient/user groups during the COVID-19 pandemic? Which groups are that and how did your work change during the COVID-19 pandemic

9. Do you work in other community settings or with community organizations, e.g., in schools, youth centers, senior centers, and others during the COVID 19 pandemic? If so, how did your work with and in the community change during the pandemic? Can you give an example?

10. What factors are supporting (could support) your commitment in community participation during the COVID-19 Pandemic, which factors are hindering it?

    Those were all our questions, thank you very much for your interesting answers. Is there something that you would like to add?
